# Better synthetic biology outcomes through balanced push-pull engineering

**DOI:** 10.1093/plphys/kiag437

**Published:** 2026-06-26

**Authors:** Blanca Jazmin Reyes-Hernández

**Affiliations:** Assistant Features Editor, Plant Physiology, American Society of Plant Biologist; Faculty of Science, Department of Plant and Environmental Sciences, Section for Plant Glycobiology, University of Copenhagen, 1871 Frederiksberg C, Denmark

Synthetic biology aims to create, control, and program cellular processes. Since emerging as a discipline in the early 2000s, the field of synthetic biology has advanced at a remarkable pace, enabling applications ranging from medicine to renewable biofuels (Cameron et al. 2014). Many synthetic biology designs and principles were first tested in microbes, which have fast generation time and enable relatively easy genetic manipulation. However, many valuable compounds remain difficult to produce in conventional microbial hosts (chassis) because they can impose a metabolic burden or even become toxic to the cells (Jongedijk et al. 2016). In this context, plants are attracting increasing interest as sustainable production platforms, owing to their natural ability to synthesize, tolerate, and store complex specialized metabolites.

In a recent study published in *Plant Physiology*, Jung and colleagues (2026) explored how the potential of plants for synthetic biology can be further enhanced. As a proof of concept, these authors focused on the production of betalains, a group of red-to-purple (betacyanins) and yellow-to-orange (betaxanthins) pigments found in crops such as beetroot, dragon fruit, and quinoa. Derived from tyrosine, betalains are valued not only for their antioxidant and potential health-promoting properties but also for their commercial importance as natural food colorants (Azeredo 2009). As demand grows for naturally derived alternatives to synthetic dyes, efficient and scalable betalain production has become increasingly desirable.

Betalain production relies on 3 enzymatic reactions that convert tyrosine into the final pigment. The enzyme P450 oxygenase CYP76AD1 first converts tyrosine into L-3,4- dihydroxyphenylalanine (L-DOPA) and can further oxidize L-DOPA into cyclo-DOPA. Alternatively, L-DOPA is converted by L-DOPA 4,5-dioxygenase (DODA) into betalamic acid, a key intermediate that combines with cyclo-DOPA to form betanidin. Finally, a glucosyltransferase (GT) catalyzes the last step, producing a stable betalain (Gandía-Herrero and García-Carmona 2013). Previous work identified 2 key regulatory points that could be targeted to enhance betalain production: tyrosine precursor and pathway flux (Lopez-Nieves et al. 2018; Grützner et al. 2021; Jung and Maeda 2024; Tan et al. 2025).

A major advance in betalain engineering came first with the development of RUBY, a construct that combines the 3 core genes, CYP76AD1, DODA, and GT. These genes are linked by 2A peptides, which mediate protein separation during translation and enable the production of multiple proteins from a single transcript. RUBYserves as a noninvasive repoter that generates a visible red pigment (He et al. 2020). Subsequent studies further enhanced this system (“pull”) by introducing the enzyme TyrA dehydrogenase (BvTyrAα) from beetroot to boost tyrosine production and strengthen betalain biosynthesis (“push”) (Fig. 1a; Jung and Maeda 2024). More recently, the eRUBY integrated both components into a single construct, increasing betalain accumulation in rice endosperm by up to 5.3-fold compared with RUBY alone, demonstrating the importance of precursor availability for maximizing pigment production (Tan et al. 2025). Despite these advances, previous work identified DODA as a remaining bottleneck in the pathway, suggesting that further gains could be achieved by balancing precursor supply with downstream pathway flux (Jung and Maeda 2024).

**Figure 1:**
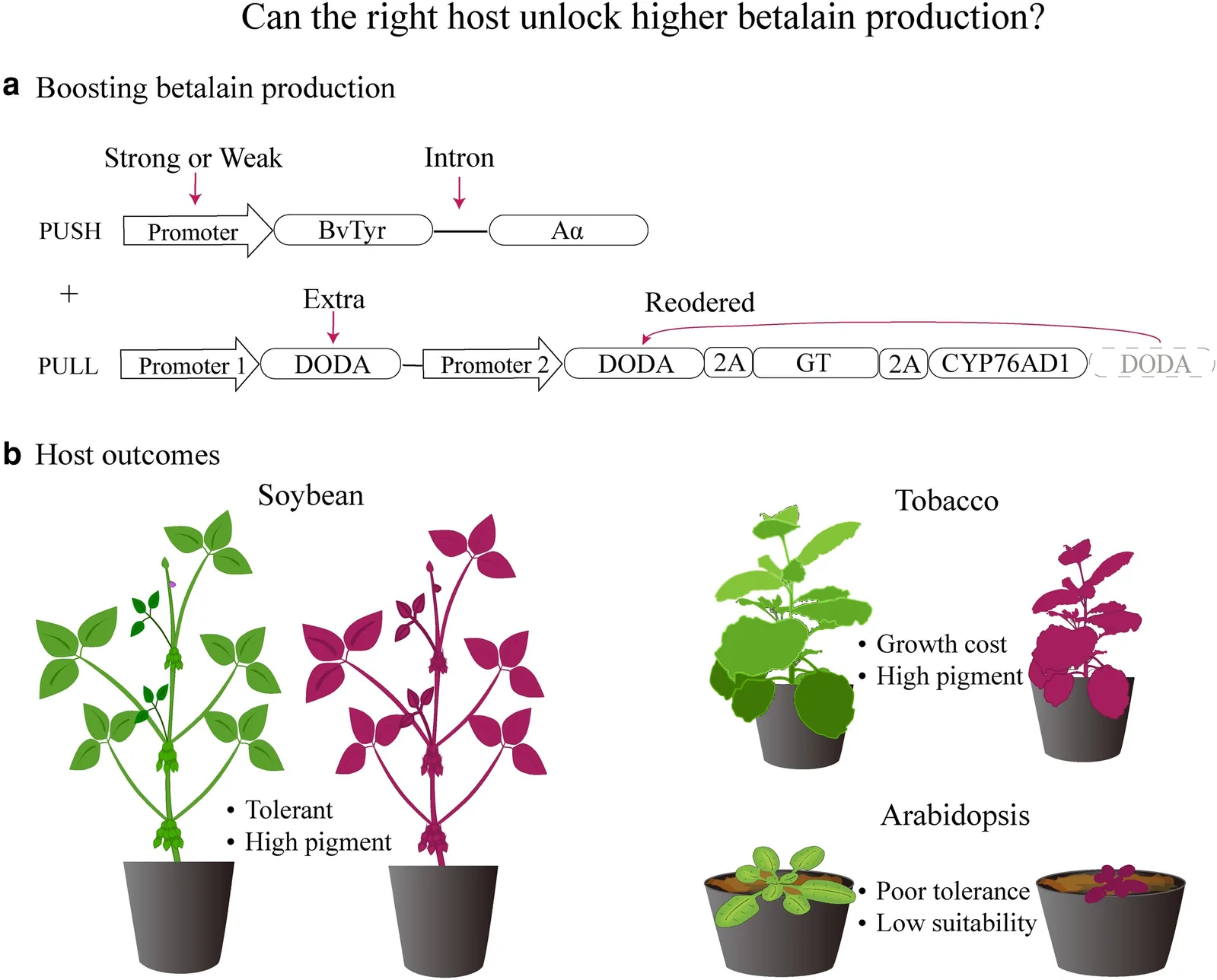
Balancing push–pull engineering and host selection for betalain production. (a) Schematic representation of the redesigned push and pull constructs used by Jung et al. (2026). The push construct (PUSH) expresses the feedback-insensitive beet TyrA dehydrogenase (BvTyrAα) under either the pAtLHB1B1 (Strong) or the pCL1(Weak) constitutive Arabidopsis promoter. BvTyrAα includes an intron to prevent expression in Agrobacterium. The construct RUBYv2 (PULL) contains an additional L-DOPA 4,5-dioxygenase (DODA) expression unit driven by the SlRbcS2 promoter (Promoter 1) and a rearranged gene order designed to enhance the pathway flux. The genes CYP76AD1, L-DOPA 4,5-dioxygenase (DODA), and glucosyltransferase (GT) expressed from the AtRbcS3 promoter (Promoter 2) are linked by 2A peptides, which enable the production of multiple proteins. Arrows indicate the main modifications introduced to optimize precursor supply and betalain biosynthesis. (b) Distinct outcomes of the same engineering strategy across plant hosts. Although betalain production was achieved in tobacco, soybean, and Arabidopsis, soybean showed the highest tolerance and sustained pigment accumulation with limited growth penalties. Tobacco accumulated high pigment levels but exhibited growth costs under Strong-PUSH conditions, whereas Arabidopsis displayed poor tolerance to the push + pull system tested by the authors. Conceptual summary based on Jung et al. (2026).

Jung and colleagues (2026) asked whether simultaneously optimizing push and pull could unlock the full potential of different plant hosts for betalain production. To address this question, as a first step, the team introduced the push and pull previously developed betalain-engineering constructs (Tan et al. 2025) into tobacco and soybean to evaluate their performance in different plants. Although stable transgenic lines were recovered for the control EV (empty vector), push, and pull constructs, no stable transformants were obtained with the push + pull construct in either species in their experiments.

To understand why the push + pull strategy failed to obtain stable transformants, the authors turned to transient expression assays in *Nicotiana benthamiana*. Metabolite analysis revealed an accumulation of L-DOPA, suggesting that precursor production exceeded the capacity of this pathway to convert it into betalains. Together with previous findings showing that maximal betalain production required an additional copy of DODA (Jung and Maeda 2024), these results pointed to an imbalance between the “push” and “pull” components of the pathway, likely interfering with the recovery of stable transgenic lines.

To try to restore the balance between precursor supply and pathway flux, the authors redesigned the push and pull components of the system. In the new pull construct, they introduced an additional DODA transcriptional unit and rearranged the order of the betalain biosynthetic genes from CYP76AD-2A-DODA-2A-GT to DODA-2A-GT-2A-CYP76AD1, placing DODA first to favor its expression. While the push construct included an intron in *BvTyrAα* to prevent any leaky expression in *Agrobacterium* (Fig. 1a).

The authors also reasoned that the strength of the push component might be critical. To test this, B*vTyrAα* was expressed under either a strong (*pAtLHB1B1*) or a weak (*pCL1*) constitutive *Arabidopsis* promoter, both active in photosynthetic tissues. Throughout this article, these configurations are referred to as “strong-push” and “weak-push,” respectively (Fig. 1a).

The redesigned constructs successfully restored the balance between precursor supply and pathway flux. In transient *N. benthamiana* assays, L-DOPA accumulated at much lower levels than with the original push/pull construct, indicating that the additional DODA copy and the redesigned RUBY (RUBYv2) cassette improved the conversion of intermediates into betalains. Consistent with this finding, the strongest betalain pigmentation was observed in the strong-push + pull combination, whereas the weak-push + pull construct produced slightly lower pigment levels. Together, these results confirmed that the new redesigned push/pull system could increase betalain production without causing excessive L-DOPA accumulation.

The team next tested whether these optimized constructs could generate stable transgenic plants. In tobacco, all constructs produced T0 lines with visible betalain pigmentation. However, the strong-push + pull plants displayed severe growth defects, whereas weak-push *+* pull plants maintained an almost normal phenotype while still accumulating high betalain levels (Fig. 1b). Notably, spectrophotometric assays revealed that betacyanin levels exceeded those naturally found in beet roots/hypocotyls. Higher levels of tyrosine and L-DOPA in strong-push + pull plants accompanied the growth defects, suggesting that excessive precursor accumulation can compromise plant fitness.

Interestingly, soybean showed a different response (Fig. 1b). Although both push + pull lines accumulated high betacyanin levels, growth defects were considerably milder than those observed in tobacco. The strongest soybean lines produced betacyanin concentrations comparable to those found in beet roots/hypocotyls while maintaining relatively normal development. Both push and push + pull lines accumulated significantly more tyrosine than wild-type plants, while L-DOPA levels were further elevated in the push + pull lines. Importantly, like tobacco, betalain pathway gene expression remained largely unchanged, indicating that increased pigment production was driven by precursor availability rather than transcriptional differences. These findings suggest that soybean is more tolerant to elevated tyrosine and L-DOPA levels and may therefore represent a more suitable chassis for betalain production.

Not all plant hosts tested by the authors responded equally to the engineered pathway. In contrast to pigmented T1 pull lines in Arabidopsis, T1 push lines showed severe growth defects. These growth defects like reticulate leaf and stunted growth, persisted in T2 and appear in T2 pull lines. Of particular interest, the push + pull constructs in T0 lines had a poor rate of transformation. Although betalain pigmentation could be achieved in few T1 push + pull lines, the strong physiological penalties, like failure to produce T2 seeds highlighted important host-dependent differences in metabolic tolerance. These findings suggest that Arabidopsis may not be an ideal chassis for high-level betalain production (Fig. 1b).

Beyond host stability, the authors also assessed the long-term stability of the engineered pathway and followed betalain production in subsequent generations. In tobacco, strong pigmentation was maintained in single insertion T1 plants, particularly in the weak-push + pull lines, which combined high betacyanin accumulation with relatively normal growth. Soybean also retained high pigment levels in the next generation, although germination and seed production remained influenced by transgene dosage and promoter strength, given that just hemizygous strong-push + pull lines germinated. Together, these results propose that efficient betalain production can be inherited, but its success depends strongly on both host species and pathway design.

Rather than identifying a universal strategy for plant metabolic engineering, this study demonstrates that the outcome of synthetic biology approaches depends on carefully matching pathway design with the metabolic capabilities of the host. Selecting the right plant chassis may therefore be just as important as optimizing the engineered pathway itself. Yet identifying the ideal host remains a major challenge, as generating and screening transgenic plants is still time-consuming and resource intensive. However, advances in automation and robotics-assisted screening could help soon to overcome this limitation (Qiande et al. 2026).

## Recent related articles in Plant Physiology:

Tan et al (2025) showed that co-expression of RUBY with a feedback-insensitive TyrA enzyme markedly enhanced betalain accumulation in rice endosperm, improving both pigment production and the antioxidant properties of the engineered tissue.

Qiande et al. (2026) showed that the BOTany Method makes plant synthetic biology more accessible by using affordable, modular robot workflows that do not require coding experience, speeding up routine cloning and molecular biology tasks, improving reproducibility.

Zhang et al. (2026) compared different R2R3-MYB regulators in a common petunia system to test how they control anthocyanin pigmentation. From a synthetic biology perspective, they used a shared host to measure how these transcription factors affect gene expression and flower color, showing that the regulators have diversified functions in pigment pathways.

## Data Availability

No additional data was used for the research described in the article.
